# Molecular characterization of the *piggyBac*-like element, a candidate marker for phylogenetic research of *Chilo suppressalis* (Walker) in China

**DOI:** 10.1186/s12867-014-0028-y

**Published:** 2014-12-17

**Authors:** Guang-Hua Luo, Xiao-Huan Li, Zhao-Jun Han, Hui-Fang Guo, Qiong Yang, Min Wu, Zhi-Chun Zhang, Bao-Sheng Liu, Lu Qian, Ji-Chao Fang

**Affiliations:** Institute of Plant Protection, Jiangsu Academy of Agricultural Sciences, Nanjing, 210014 China; Education Ministry Key Laboratory of Integrated Management of Crop Diseases and Pests, College of Plant Protection, Nanjing Agricultural University, Nanjing, 210095 China; Jiangsu Entry-Exit Inspection and Quarantine Bureau, Nanjing, 210001 China

**Keywords:** Transposon, piggyBac, Molecular characterization, Evolution, *Chilo suppressalis*

## Abstract

**Background:**

Transposable elements (TEs, transposons) are mobile genetic DNA sequences. TEs can insert copies of themselves into new genomic locations and they have the capacity to multiply. Therefore, TEs have been crucial in the shaping of hosts’ current genomes. TEs can be utilized as genetic markers to study population genetic diversity. The rice stem borer *Chilo suppressalis* Walker is one of the most important insect pests of many subtropical and tropical paddy fields. This insect occurs in all the rice-growing areas in China. This research was carried out in order to find diversity between *C. suppressalis* field populations and detect the original settlement of *C. suppressalis* populations based on the *piggyBac*-like element (PLE). We also aim to provide insights into the evolution of PLEs in *C. suppressalis* and the phylogeography of *C. suppressalis*.

**Results:**

Here we identify a new *piggyBac*-like element (PLE) in the rice stem borer *Chilo suppressalis* Walker, which is called *CsuPLE1.1* (GenBank accession no. JX294476). *CsuPLE1.1* is transcriptionally active. Additionally, the *CsuPLE1.1* sequence varied slightly between field populations, with polymorphic indels (insertion/deletion) and hyper-variable regions including the identification of the 3′ region outside the open reading frame (ORF). *CsuPLE1.1* insertion frequency varied between field populations. Sequences variation was found between *CsuPLE1* copies and varied within and among field populations. Twenty-one different insertion sites for *CsuPLE1* copies were identified with at least two insertion loci found in all populations.

**Conclusions:**

Our results indicate that the initial invasion of *CsuPLE1* into *C. suppressalis* occurred before *C. suppressalis* populations spread throughout China, and suggest that *C. suppressalis* populations have a common ancestor in China. Additionally, the lower reaches of the Yangtze River are probably the original settlement of *C. suppressalis* in China. Finally, the *CsuPLE1* insertion site appears to be a candidate marker for phylogenetic research of *C. suppressalis*.

**Electronic supplementary material:**

The online version of this article (doi:10.1186/s12867-014-0028-y) contains supplementary material, which is available to authorized users.

## Background

Transposable elements (TEs, transposons) are mobile genetic DNA sequences, and they are found in the genomes of nearly all eukaryotes [1,2]. TEs can insert copies of themselves into new genomic locations and they have the capacity to multiply. Therefore, TEs make up a significant portion of the eukaryotic genome and have driven genome evolution in many ways, including gene expression alterations, gene deletions and insertions, chromosome rearrangements and others [3-6]. TEs are divided into two major classes based on their transposition intermediate and distinct structural features [7]. Class I TEs, which are also called retrotransposons, use a “copy-and-paste” mechanism that involves an RNA intermediate. This intermediate is reverse transcribed before its reintegration into a new position. Class II TEs, which are also called DNA transposons, use a DNA-mediated mode of “cut-and-paste” transposition.

The *piggyBac* element, which is a class II transposon, was originally discovered in the TN-368 cell line of the cabbage looper moth *Trichoplusia ni* [8,9]. It transposes via a “cut-and-paste” mechanism, inserting exclusively at 5′-TTAA-3′ tetranucleotide target sites and excising with precision, leaving no footprint [10]. Transposons similar to the original functional *piggyBac* IFP2 called *piggyBac*-like elements (PLEs) have been found in diverse organisms, including fungi, plants, insects, crustaceans, urochordates, amphibians, fishes and mammals [1,11-15]. PLEs are highly divergent and can be classified into three main classes, namely by high sequence similarity to *IFP2*, moderate sequence similarity to *IFP*2 and very distantly related ancient elements [14].

The rice stem borer *Chilo suppressalis* Walker is one of the most important insect pests of many subtropical and tropical paddy fields in Asia, North Africa and southern Europe. This insect occurs in all the rice-growing areas in China, and it colonizes a wide range of hosts such as rice (*Oryza sativa*), water-oat (*Zizania aquatica*) and chufa (*Eleocharis tuberosa*) [16]. It is assumed that all *C. suppressalis* field populations in China have a common ancestor. However, there is no clear evidence of this. We want to know if *C. suppressalis* field populations have a common ancestor, and if so, where this common ancestor originated.

For this paper, we isolated a group of endogenous PLEs from the *C. suppressalis* genome, which were designated as *CsuPLE1*s. The *CsuPLE1* copy with an intact open reading frame (ORF) was named *CsuPLE1.1*. The frequency of *CsuPLE1.1* insertion at a specific locus in the *C. suppressalis* genome varied among populations. This study will contribute to our understanding of the distribution and characteristics of the *piggyBac* family. In addition, the analysis *CsuPLE1*s sequence variants identified in *C. suppressalis* from different field populations provides insights into the evolution of *CsuPLE1*s. Based on the insertion sites and sequence variations of *CsuPLE1*s, the phylogeography of *C. suppressalis* is discussed.

## Results

### Characterization of *piggyBac*-like element (PLE) in *C. suppressalis*

**Figure 1:**
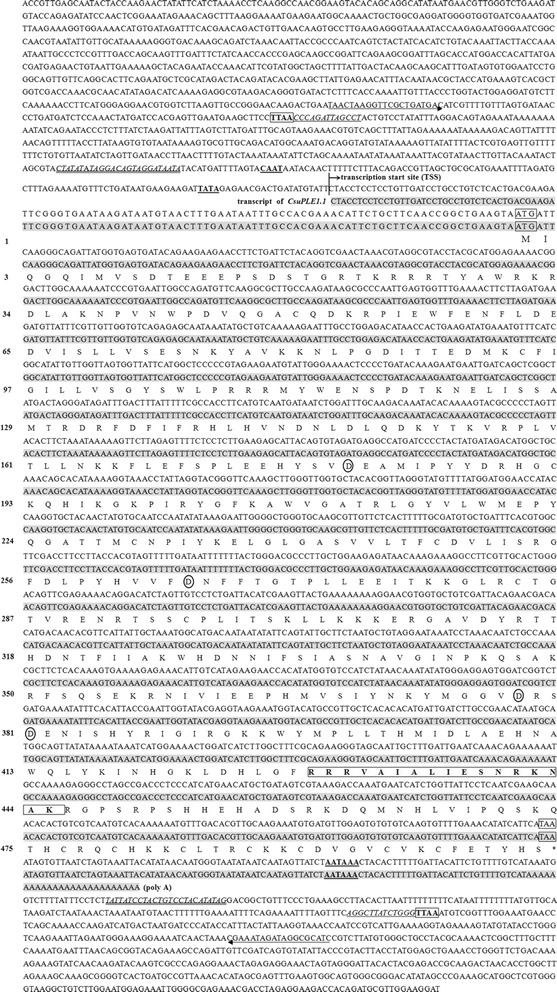
**Nucleotide sequence, transcript and putative transposase of the** ***CsuPLE1.1*** **.** The nucleotide sequence in the light gray shadow is the *CsuPLE1.1* transcript. The inverted terminal repeats (ITRs) are underlined and italicized. The sub-terminal inverted repeats are double-underlined and italicized. The 4 bp (TTAA) target site duplications (TSDs) are boxed and in bold. Putative CAAT, TATA and AATAAA polyadenylation signal site sequences are underlined and in bold. The primers used in flanking PCR are indicated by left and right arrows below the corresponding sequences. The “DDD-motif” amino acid residues in the putative transposase are indicated by open circles. The bipartite nuclear localization signal (NLS) is boxed and in bold.

**Figure 2:**
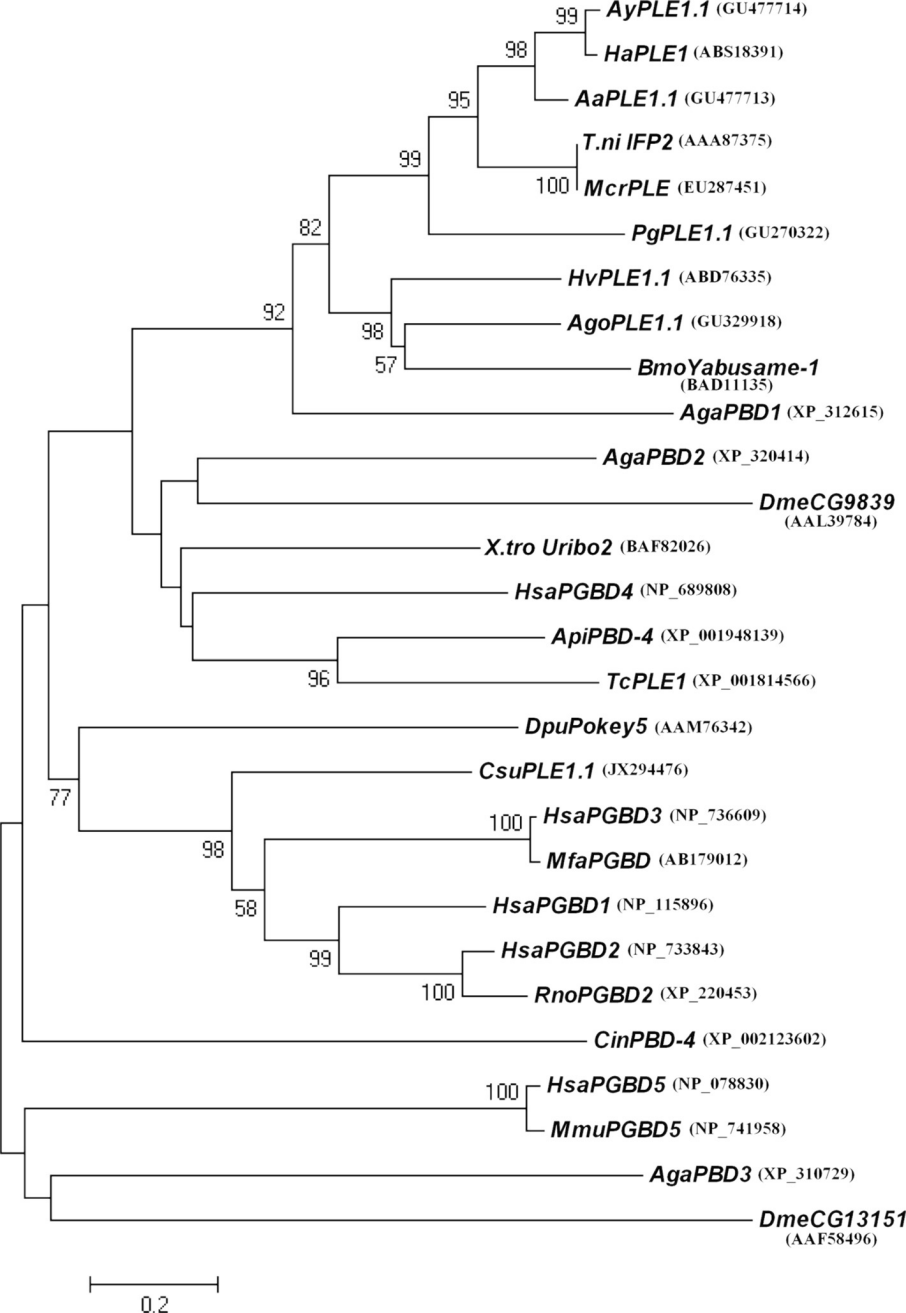
**Phylogenetic relationships among** ***piggyBac*** **-like element transposase amino acid sequences.** The tree was generated by the neighbor-joining method. The numbers at the nodes are the bootstrap values (>50%) for 5000 replications. The GenBank accession numbers are in brackets. Abbreviations: *Aa*, *Argyrogramma agnata*; *Aga*, *Anopheles gambiae*; *Ago*, *Aphis gossypii*; *Api*, *Acyrthosiphon pisum*; *Ay*, *Agrotis ypsilon*; *Bmo*, *Bombyx mori*; *Cin*, *Ciona intestinalis*; *Csu*, *Chilo suppressalis*; *Dme*, *Drosophila melanogaster*; *Dpu*, *Daphnia pulicaria*; *Ha*, *Helicoverpa armigera*; *Hsa*, *Homo sapiens*; *Hv*, *Heliothis virescens*; *Mcr*, *Macdunnoughia crassisigna*; *Mfa*, *Macaca fascicularis*; *Mmu*, *Mus musculus*; *Pg*, *Pectinophora gossypiella*; *Rno*, *Rattus norvegicus*; *Tc*, *Tribolium castaneum*; *T.ni*, *Trichoplusia ni*; *X.tro*, *Xenopus tropicalis*.

A full-length PLE from *C. suppressalis* was obtained and named *CsuPLE1.1* (GenBank accession no. JX294476, Figure 1). This PLE is 2406 bp in length and contains all the characteristic structures of a PLE, including 13 bp inverted terminal repeats (ITRs), asymmetrically located 25 bp sub-terminal inverted repeats and a single open reading frame (ORF) encoding a transposase of 505 amino acids. The putative transposase contains all the aspartate residues of the “DDD” motif, which correspond to D268, D346, D447 and D450 in *T. ni IFP2* transposase. As in other PLEs, the *CsuPLE1.1* was inserted into typical tetranucleotide target-site TTAA duplications and flanked by a sequence (912 bp at 5′-end and 576 bp at 3′) that was not significantly homologous to any gene sequences in the GenBank. Notably, *CsuPLE1.1* also has a putative CAAT site, and a TATA site exists at nt 392–395 and nt 478–481. There is also a polyadenylation signal site at nt 2188–2193, which is characteristic of an actively translated protein. Alignments showed that, among the known PLEs, the putative transposase of *CsuPLE1.1* shared the highest similarity (53%) with *HsaPGBD3* transposase (Figure 2), and belongs to a class that is moderately similar to IFP2.

RACE amplification using a cDNA template revealed that *CsuPLE1.1* was expressed as a 1748 bp transcript with a 111 bp 5′ untranslated region (UTR) and a 119 bp 3′ UTR containing a 24 bp poly (A) tail (Figure 1).

### Insertion site, TSDs and ITR variations

The 5′ TE display showed that the insert sites of *CsuPLE1*s varied between populations (Additional file 1: Figure S1). Further sequencing results obtained 21 different insertion sites among 72 flanking sequences from 12 field populations (Table 1 and Additional file 2: Table S1). Two insertion sites were found in all populations (insertion sites 1 and 2). One insertion site was found in nine populations (insertion site 8). One insertion site was found in seven populations (insertion site 7). One insertion site was found in five populations (insertion site 3). Two insertion sites were found in four populations (insertion sites 4 and 14). One insertion site was found in three populations (insertion site 9) and the remaining 13 insertion sites were found in only one or two populations. Almost one half of all populations had a unique insert site, found only in that population (Table 1).

**Table 1:** **5′ insertion sites of** ***CsuPLE1*** **in** ***C. suppressalis*** **genome**

**5′ Insertion site**	**Sampling locations**											
	**GY**	**JJ**	**XY**	**SY**	**JZ**	**DY**	**LS**	**GZL**	**HX**	**TC**	**YJ**	**YX**
**Site 1**	√	√	√	√	√	√	√	√	√	√	√	√
**Site 2**	√	√	√	√	√	√	√	√	√	√	√	√
**Site 3**	√	√	×	×	×	√	√	×	×	×	√	×
**Site 4**	√	√	×	×	×	×	×	×	√	×	√	×
**Site 5**	√	×	×	×	×	×	×	×	×	×	×	×
**Site 6**	√	×	×	×	×	×	×	×	×	×	×	×
**Site 7**	√	×	×	×	×	√	√	√	√	√	×	√
**Site 8**	√	√	√	√	√	√	×	√	√	×	×	√
**Site 9**	×	√	×	×	×	√	√	×	×	×	×	×
**Site 10**	×	×	√	×	×	×	×	×	×	×	×	×
**Site 11**	×	×	×	√	×	×	×	×	×	×	×	×
**Site 12**	×	×	×	√	×	×	×	×	×	×	×	×
**Site 13**	×	×	×	√	×	×	×	√	×	×	×	×
**Site 14**	×	×	×	×	√	×	√	×	×	√	√	×
**Site 15**	×	×	×	×	×	√	×	×	×	×	×	×
**Site 16**	×	×	×	×	×	×	√	×	×	×	×	×
**Site 17**	×	×	×	×	×	×	√	√	×	×	×	×
**Site 18**	×	×	×	×	×	×	×	√	√	×	×	×
**Site 19**	×	×	×	×	×	×	×	×	√	×	×	×
**Site 20**	×	×	×	×	×	×	×	×	×	√	×	×
**Site 21**	×	×	×	×	×	×	×	×	×	×	√	×

The presence and absence of each insertion site within each population is indicated by a **√** and × respectively.

Among these 21 different insertion sites, most of the insertions occurred at a TTAA target site, which is characteristic of the TTAA-specific family of *piggyBac* transposons. Only four target site duplications (TSDs) contained variations. In insertion site 5, the TSD was CTAT; In insertion site 9 and 16, the TSDs were ATAT; In insertion site 10, the TSD was CTAA. The ITR analysis showed that the 13 bp ITRs of CsuPLE1 were conserved in most individuals, with only two ITRs containing slight variations. In insertion site 14, there was a C-A variation in the ITRs; In insertion site 16, there was a G-A variation in the ITRs (Table 2).

**Table 2:** **The variations of 5′TSDs and 5′ITRs of** ***CsuPLE1*** **s**

**Insertion site**	**5′ TSDs**	**5′ ITRs**
1	TTAA	5′-CCCAGATTAGCCT
2	TTAA	5′-CCCAGATTAGCCT
3	TTAA	5′-CCCAGATTAGCCT
4	TTAA	5′-CCCAGATTAGCCT
5	*C*TA*T*	5′-CCCAGATTAGCCT
6	TTAA	5′-CCCAGATTAGCCT
7	TTAA	5′-CCCAGATTAGCCT
8	TTAA	5′-CCCAGATTAGCCT
9	*A*TA*T*	5′-CCCAGATTAGCCT
10	*C*TAA	5′-CCCAGATTAGCCT
11	TTAA	5′-CCCAGATTAGCCT
12	TTAA	5′-CCCAGATTAGCCT
13	TTAA	5′-CCCAGATTAGCCT
14	TTAA	5′-*A*CCAGATTAGCCT
15	TTAA	5′-CCCAGATTAGCCT
16	*A*TA*T*	5′-CCCA*A*ATTAGCCT
17	TTAA	5′-CCCAGATTAGCCT
18	TTAA	5′-CCCAGATTAGCCT
19	TTAA	5′-CCCAGATTAGCCT
20	TTAA	5′-CCCAGATTAGCCT
21	TTAA	5′-CCCAGATTAGCCT

The variant nucleotides are in italics.

### *CsuPLE1.1* insertion frequency and sequence variations

Flanking PCRs for testing the presence or absence of *CsuPLE1.1* insertions were performed on 45 randomly collected individuals from the 21 populations. The frequencies of individuals with the insertion varied between populations (Table 3). From the 945 individuals tested, 384 were heterozygous for the *CsuPLE1.1* insertion. All remaining individuals did not have a *CsuPLE1.1* insertion.

**Table 3:** **The frequency of** ***CsuPLE1.1*** **insertion in each field population**

**Rank of** ***CsuPLE1.1*** **insertion frequency**	**Sampling locations**	**Frequency of** ***CsuPLE1.1*** **insertion (mean ± SEM)**
1	YX	0.8 ± 0.0387
2	HX	0.6667 ± 0.0771
3	GZL	0.578 ± 0.0588
3	SY	0.578 ± 0.089
5	GY	0.511 ± 0.022
5	YZ	0.511 ± 0.097
7	TC	0.489 ± 0.097
8	QC	0.4667 ± 0.0384
9	JZ	0.4443 ± 0.0588
10	GX	0.3557 ± 0.0802
10	LH	0.3557 ± 0.0588
12	DY	0.3333 ± 0.0771
12	NC	0.3333 ± 0.0667
12	MH	0.3333 ± 0.0384
15	JJ	0.311 ± 0.0588
16	FN	0.289 ± 0.0443
16	YS	0.2887 ± 0.0802
18	YJ	0.2667 ± 0.0667
19	XY	0.222 ± 0.0588
19	GN	0.222 ± 0.0588
21	LS	0.1777 ± 0.0447

**Figure 3:**
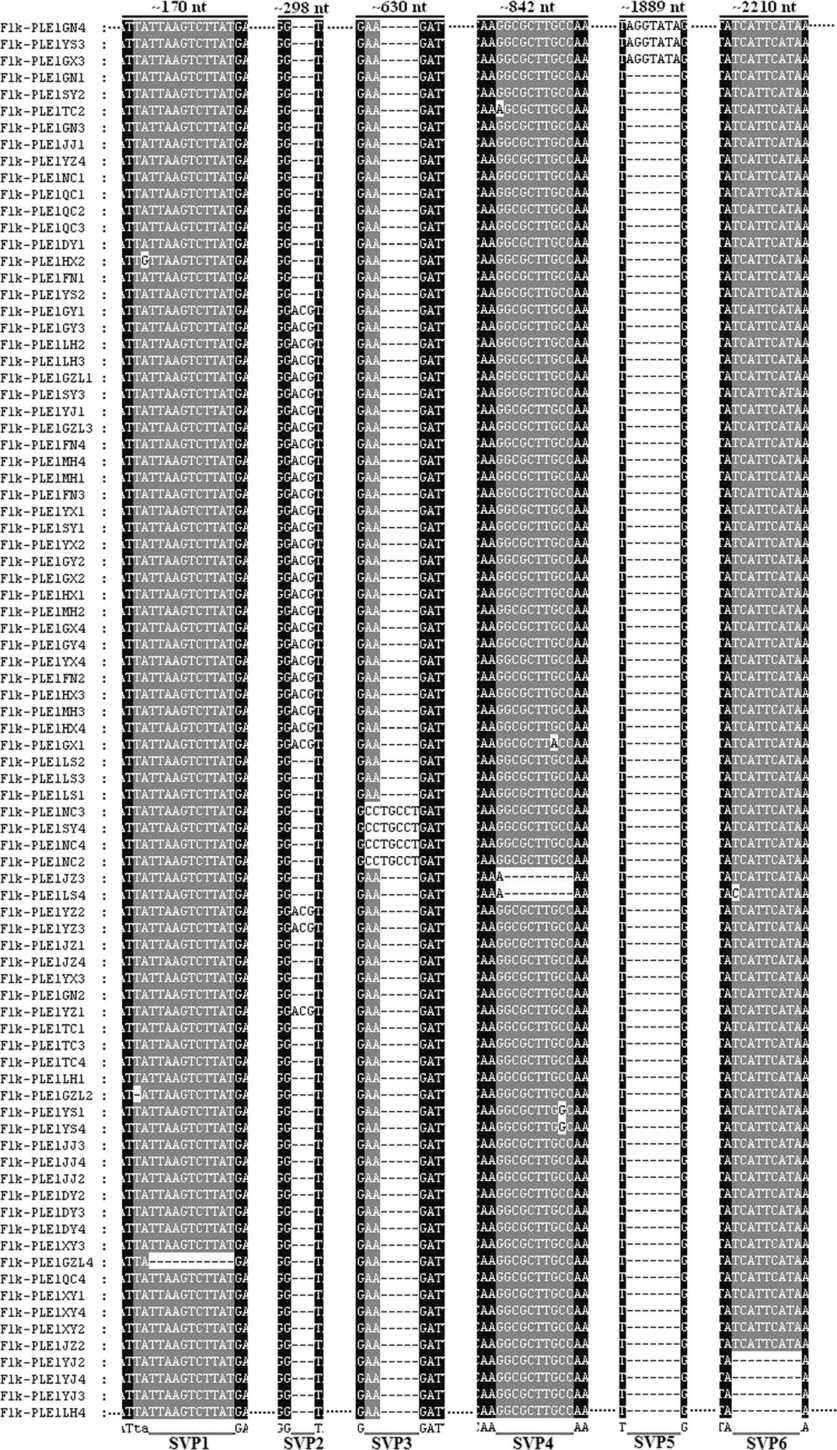
**Six special variation positions (SVP).** The location of each SVP in the sequence is indicated by Arabic numbers on the top. The omitted sequences are indicated by ellipses. The black short line indicates that there is no base. Flk-PLE1GN is from the Guangning (GN) population. Flk-PLE1YS is from the Yangshuo (YS) population. Flk-PLE1GX is from the Ganxian (GX) population, and so on.

A total of 84 copies of the *CsuPLE1.1* insertion were cloned from 84 individuals across the 21 field populations. These sequences share high levels of similarity with the exception of the *Flk-PLE1JZ*4 copy, which has an approximately 130 bp sequence deletion. There were substitutions, deletions and/or insertions in each copy (Additional file 3: Table S2). Furthermore, in some *CsuPLE1.1* copies, there were indels of three or more bases. We defined this position as special variation position (SVP). Six SVPs were identified (Figure 3). SVP1 from copy *Flk-PLE1GZL*4 has an 11 bp deletions at nt ~170. In SVP3, the copies *Flk-PLE1NC*2, *Flk-PLE1NC*3, *Flk-PLE1NC*4 and *Flk-PLE1SY*4 have 2 bp deletions and 7 bp insertions at nt ~630. In SVP4, the copies *Flk-PLE1JZ*3 and *Flk-PLE1LS*4 have 9 bp deletions at nt ~842. In SVP5, the copies *Flk-PLE1GX*3, *Flk-PLE1YS*3 and *Flk-PLE1GN*4 have 7 bp insertions at nt ~1889. In SVP6, the copies *Flk-PLE1YJ*2, *Flk-PLE1YJ*3, *Flk-PLE1YJ*4 and *Flk-PLE1LH*4 have 9 bp deletions at nt ~2210. Three bases, ACG, were found in only some PLE1 copies particularly at nt ~298 of SVP2.

Among the 84 sequences, 63 sequences have a putative intact ORF. These 63 sequences shared a high degree of sequence similarity. While, position nt ~2310, in the 3′ region outside of the ORF, showed the highest nucleotide variability between *CsuPLE1.1* copies. This site, which we called the variation hotspot, contained a number of indels (Additional file 4: Figure S2).

The variation rate (*Rv*) of the *CsuPLE1.1* copies differed inside and outside the ORF region. The variation rates inside the ORF region and in the PLE 5′ region outside the ORF were significantly lower than the PLE 3′ outside the ORF region (Table 4).

**Table 4:** **The variation rate in different areas of** ***CsuPLE1.1***

**Area**	**Length (average ± SEM)**	No. of position variances* (average ± SEM)	Variation rate* (average ± SEM)
5′ outside the ORF region	613.2 ± 0.3	3.8 ± 0.4	0.0062 ± 0.0007 B
Inside the ORF region	1518 ± 0	8 ± 0.8	0.0053 ± 0.0005 B
3′ outside the ORF region	278 ± 0.1	3.1 ± 0.3	0.0113 ± 0.0011 A

*A certain position contains an/a insertion, deletion, transition or transversion was recorded as one variance position. For the variation rate, were significant variations (p < 0.01) between the 3′ outside the ORF region and inside the ORF region, and the 5′ outside the ORF region.

### Phylogenetic tree of *C. suppressalis* field populations

A MP phylogenetic tree and a UPGMA phylogenetic tree were constructed independently. In the MP phylogenetic tree, there were many clusters and the phylogenetic relationship between each was ambiguous (Additional file 5: Figure S3). In the UPGMA tree, there were three clusters: JZ, GY and XY formed one clade; YJ, LS, JJ and DY formed a second clade; and TC, SY, HX, GZL and YX formed a third clade (Additional file 6: Figure S4).

## Discussion

Since *piggyBac* is one of the most popular transposons used for transgenesis, searching for new active PLEs has attracted lots of attention. However, only a few active PLEs have been reported to date, including *IFP2*, *Uribo2*, *McrPLE* and *AgoPLE1.1* [15,17-19]. Here we identified another potentially active PLE. This PLE has the intact structure of a *piggyBac* transposon, including TTAA insertion sites, 13 bp ITRs, 25 bp subterminal inverted repeats, and a single ORF encoding a transposase of 505 amino acids with a perfect “DDD-motif”. The transposase was also shown to be transcriptionally active with a 1748 bp transcript cloned from *C. suppressalis*.

There was a high degree of sequence similarity in *CsuPLE1*s from different field populations. All the tested populations shared two identical insertion sites, and each population also had their own unique insert sites. Since all inactive copies of TE will be fixed or lost in the population over time if they are neutral, insertion sites will become more homogeneous in populations over time [20,21]. Our results therefore suggest that a few *CsuPLE1* copies in *C. suppressalis* may be functional and still moving. Investigators have previously hypothesized that if TEs with high sequence similarities could maintain their original structure in their hosts, then the invasion of the TE was a recent event [15,22,23]. The high sequence similarities between *CsuPLE1* copies found in this study suggest that the invasion of *CsuPLE1* was a recent event.

The transposition activities of intact transposons are often regulated or silenced at the transcriptional, translational, or transpositional level for the survival of transposons and their hosts [6,24-26]. As a result, there are many transposable elements with mutations or variations within their host organisms [27-29]. Sequence mutations may be randomly distributed in transposons. However our results indicate that the 3′ region outside the ORF in the *CsuPLE1* transposons sequence had the highest variation rates. A variation hotspot was also found in this 3′ region. Generally, regions with high numbers of mutation are the result of complex cellular processes including (i) interactions between DNA and mutagens, (ii) repair of premutational lesions, (iii) local reduction in the fidelity of DNA polymerization, and (iv) expression and selection of a protein (RNA) molecule from which mutations have been detected [30,31]. This region is complex and deserves further study.

Of the 21 insertion sites found, two occurred in all field populations and five occurred in multiple field populations. Thus, these results imply that *CsuPLE1* existed in *C. suppressalis* prior to the expansion of the insect host populations into new regions. Meng et al. stated that *C. suppressalis* had strong population structure with three genetic clusters, i.e. a central China (CC) clade, a northern plus northeastern China (NN) clade and a southwestern China (SW) clade [32]. In these three clades, *C. suppressalis* had arisen from separate refuges and experienced parallel evolution. However, our results suggest that Chinese *C. suppressalis* populations have a common ancestor. The research of retrotransposon Ty3/gypsy in *C. suppressalis* shown that one insertion site of Ty3/gypsy existed in all *C. suppressalis* filed populations [33]. This also supports our conclusion that *C. suppressalis* populations have a common ancestor in China.

The MP phylogenetic tree showed that many small clusters. This was due to the high sequence similarity. However, in the 84 *CsuPLE1.1* copies, there were six SVPs. Based on our results and Meng et al.’s findings [32], we conclude that the *C. suppressalis* populations of SY, GX, YS and GN belong to the central China (CC) clade; and the *C. suppressalis* populations in JZ and LS belong to the southwestern China (SW) clade.

In the UPGMA phylogenetic tree, three clades were found. In the first clade, JZ and XY come from similar geographic areas. However, they are far from GY population. In the second clade, LS, JJ and DY come from the same geographic (the Sichuan Basin). YJ belongs to coastal areas, with a similar temperature, humidity and seasonal temperature difference to the Sichuan Basin. In the third clade, TC and HX is close to each other and have similar environmental conditions. However, SY, GZL and YX are far from each other, and have different environments. This result is not entirely consistent with Meng et al.’s result. This may be due to our small sample size or may be because our choice of method reveals a different phylogenetic relationship between *C. suppressalis* populations. Our research indicated that the insertion sites were a candidate marker for phylogenetic research of *C. suppressalis*.

**Figure 4:**
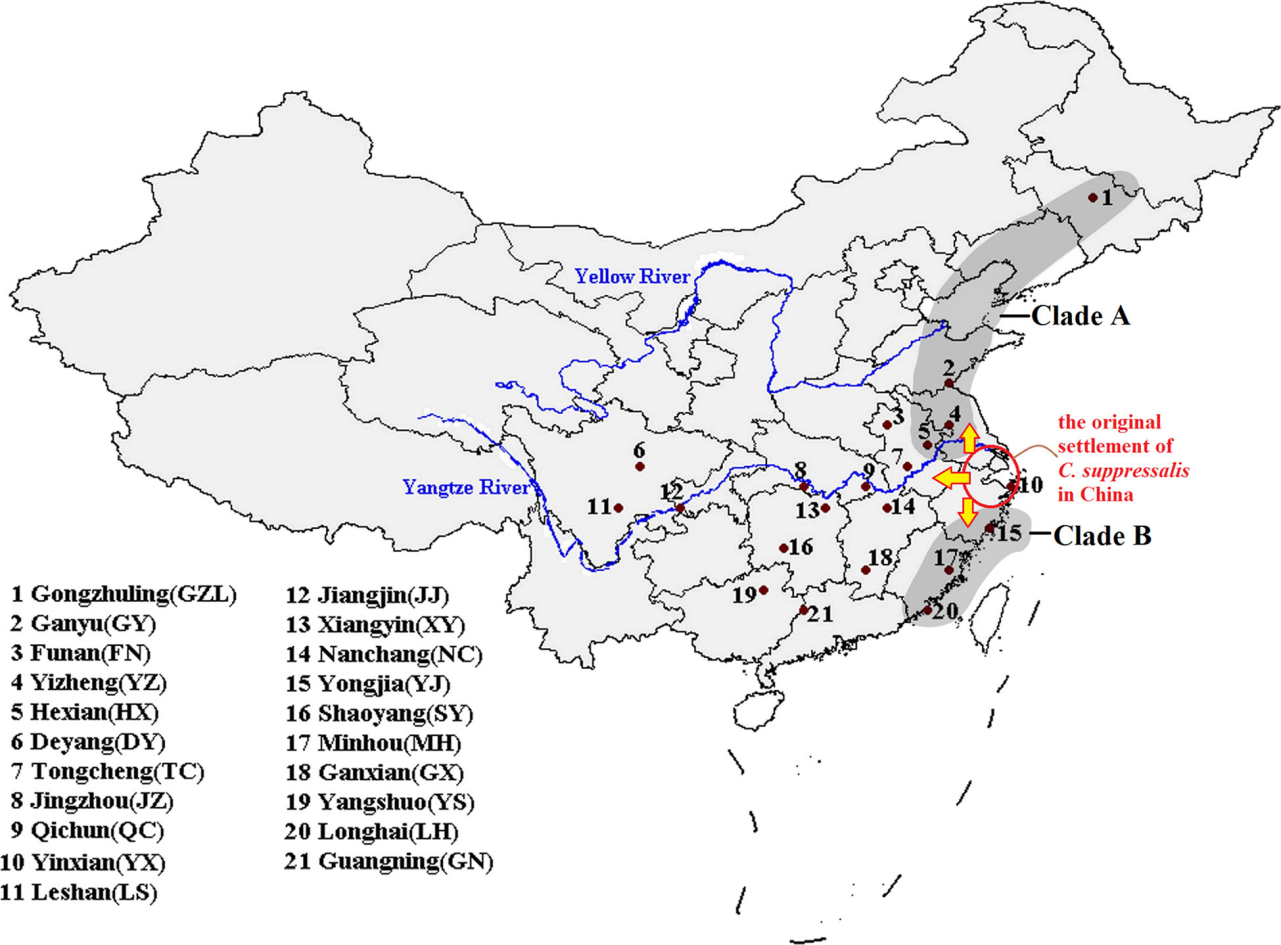
**Sampling locations and potential migration directions of** ***C. suppressalis*** **.** The letters in the brackets are the location abbreviations. The putative original settlement of *C. suppressalis* in China is indicated by red circle. The putative migration directions of *C. suppressalis* are indicated by yellow arrows.

Rice is the main host plant for *C. suppressalis*. Gene flow in *C. suppressalis* follows a similar pattern to the expansion of rice domestication in China [32]. It has been suggested that the lower reaches of the Yangtze River in China were the first rice farming region, although there are debates about the origin of rice [34-36]. Meng et al. found gene flow of *C. suppressalis* in CC and SW regions tends to move west. In the CC region this is from Ningbo towards regions such as Quzhou, Nangchang, and in the SW region, this is from Liuzhou towards regions such as Guiyang and Yaan. In the NN region gene flow moves northward from Wuhan or Zhumadian to Changchun [32]. These results together suggest that the lower reaches of the Yangtze River are probably the original settlement of *C. suppressalis* in China. Our results have shown that the *CsuPLE1.1* insertion frequency was the highest in YX populations, located in the lower reaches of the Yangtze River. Moreover, the frequency of *CsuPLE1.1* insertions decreases with increasing distance from YX. If the lower reaches of the Yangtze River are the original settlement of *C. suppressalis* in China, we proposed that the *CsuPLE1.1* invasion event initially occurred at the lower reaches of the Yangtze River. As the transposition of transposons can help host organisms adapt, we suggest that the *CsuPLE1.1* had more transpositional opportunities as *C. suppressalis* expanded into new areas and new environments. The *CsuPLE1.1* insertion frequencies in Clade A (including GZL, GY, YZ and HX populations) were higher than in Clade B (including YJ, MH and LH populations) (Figure 4 and Table 3). This may be due to fewer *C. suppressalis* generations each year in Clade A (2 ~ 3 generations per year) compared to Clade B (3 ~ 4 generations per year) and therefore less opportunity for *CsuPLE1.1* transposition in Clade A. Another reason for differences in insertion frequency could be differences in environmental stress. For example, the *CsuPLE1.1* insertion frequency in FN population was lower than all other nearby populations (Figure 4 and Table 3). In FN, upland rice, which has lower nutrient levels than non-upland rice, was planted. Our previous research showed that the average individual body weight of *C. suppressalis* in FN was lighter than other field population [37]. Also, winters in FN are colder and drier than other nearby populations. *C. suppressalis* in FN therefore faces greater challenges to survive and such stress potentially provides more transpotition opportunity for the *CsuPLE1.1* in this population.

## Conclusions

*C. suppressalis* occurs in all rice-growing areas in China, and they are non-migratory insects. Based on our results, we suggest that *C. suppressalis* populations have a common ancestor in China. The initial invasion of *CsuPLE1* in *C. suppressalis* occurred before *C. suppressalis* populations spread throughout China, and the invasion of *CsuPLE1* transposons was a recent event. Additionally, the lower reaches of the Yangtze River are probably the original settlement of *C. suppressalis* in China. Moreover, the insertion sites of *CsuPLE1s* should be a candidate marker for the phylogenetic research of *C. suppressalis*.

## Methods

### Sample collection and DNA isolation

The *C. suppressalis* samples were collected from 21 paddy rice field locations in China (Figure 4 and Additional file 7: Table S3), and were kept at −80°C until DNA extraction. Forty-five individual samples were randomly picked from each field population, and genomic DNA (gDNA) was prepared using an AxyPrep DNA Extraction Kit (Axygen Biosciences, Hangzhou, China) by following the protocol provided by the manufacturer.

### PCR amplification and sequence analysis

In order to obtain intact sequences of PLEs from *C. suppressalis*, the transcriptome of *C. suppressalis* was surveyed and a putative PLE fragment with the longest sequence (about 1700 bp) was found. Based on this sequence, one pair of specific primers was designed (SPF1: 5′-TGTATTCTACCTCCTCCTGTTG-3′; SPR1: 5′- AAAACACTTGACACACACTCCA-3′). Using these specific primers, a 1616 bp fragment of *CsuPLE1* was amplified from Hexian (HX) individual samples by PCR. The purpose of this PCR was to verify the PLE fragment sequence originating from the transcriptome of *C. suppressalis*. The PCR was performed using *LA Taq* polymerase (TaKaRa Biotechnology, Dalian, China) with the following protocol: 95°C for 3 min; 94°C for 30 s, after which the annealing temperature of the reaction was decreased by 1°C for 30 s every cycle, from 60°C to 50°C, followed by 72°C for 1 min 40 s; 26 cycles of 94°C for 30 s, 53°C for 30 s, and 72°C for 1 min 40 s; and final elongation at 72°C for 10 min. The final PCR volume was 25 μl containing approximately 50 ng gDNA, 0.2 mM of each dNTP, 1.5 mM of Mg2+, 0.2 μM of each primer, 2.5 μl of 10 × *LA* PCR buffer (Mg2+ free) and 0.25 μl (5 U/μl) of *LA Taq* polymerase (TaKaRa).

Based on this 1616 bp fragment, two pairs of nested primers for inverse PCR were designed. Inverse PCR was then performed on HX individuals with these two pairs of nested primers to obtain the full-length *CsuPLE1*. The following two pairs of primers were used for the nested inverse PCR: external primer pair (IPS1: 5′-TTGGCTTTCGCAGAAGGGTA-3′ and IPA1: 5′-CGTTTTCTCCATGCGTAGGTA-3′); Internal primer pair (IPS2: 5′-CATGCTGATAGTCGTAAAGACCA-3′ and IPA2: 5′-ATCACTCACCATAATCTGCCCT-3′). The inverse PCR was performed using LA Taq polymerase (TaKaRa) with the following protocol: 95°C for 3 min; 94°C for 30 s, after which the annealing temperature of the reaction was decreased by 1°C for 30 s every cycle, from 63°C to 53°C, followed by 72°C for 4 min; 25 cycles of 94°C for 30 s, 53°C for 30 s, and 72°C for 4 min; and final elongation at 72°C for 10 min. The final PCR volume is 25 μl containing approximately 50 ng gDNA, 0.2 mM of each dNTP, 1.5 mM of Mg2+, 0.2 μM of each primer, 2.5 μl of 10 × LA PCR buffer (Mg2+ free) and 0.25 μl (5 U/μl) of *LA Taq* polymerase (TaKaRa).

All PCR products were purified with an AxyPrep DNA Gel Extraction Kit (Axygen) and directly cloned into the pGEM-T Easy vector (Promega, Madison, WI, USA), and three clones were sequenced by GenScript Biotechnology Co., Ltd. Nanjing, China. The sequencing results were compared with non-redundant databases in the NCBI server using BLASTX and TBLASTX (http://blast.ncbi.nlm.nih.gov/Blast.cgi). CLUSTAL X1.8 [38] was used to align the putative transposase sequence of *CsuPLE1* to 27 PLEs with full-length transposases from other species sourced from Genbank. Phylogenetic analysis of the different PLE transposases was then performed on the aligned sequences in MEGA version 4 using the neighbor-joining method [39].

### RNA extraction and first-strand cDNA synthesis for RT-PCR

*Chilo suppressalis* individuals from our laboratory strain were chosen at random for total RNA extraction. One forth-instar larvae was stored at −80°C for subsequent RNA extraction. Total RNA was isolated using a Promega SV Total RNA Isolation system (Promega) using the manufacturer’s protocol.

Approximately 1 μg of total RNA was used as a template for first-strand cDNA synthesis with the PrimeScript RT reagent kit (TaKaRa) in a 20 μl reaction. Reactions were conducted at 37°C for 15 min, followed by 85°C for 5 s, and stopped by cooling on ice for 5 min.

### Determination of *CsuPLE1* transcript by RACE

In order to determine the sequence of the intact transcript of *CsuPLE1*, 5′- and 3′-RACE was conducted using the SMART RACE cDNA Amplification Kit (Clontech, Mountain View, CA, USA) using the manufacturer’s protocol. The first-strand 5′-RACE-ready cDNA and 3′-RACE-ready cDNA was synthesized from 1 μg of total RNA using SMARTScribe Reverse Transcriptase (Clontech). The synthesized first-stand 5′-RACE-ready cDNA was used as a template to amplify the 5′ end of *CsuPLE1* cDNA using the Universal Primer A Mix (UPM) and the Nested Universal Primer A (NUP) with the two *CsuPLE1*-specific reverse primers (5RACE01: 5′ CTCCAGGCAAATTCTTTTTGACAGCA-3′ and 5RACE02: 5′- TCAAACCACTCAATTGGGCGCTTATC-3′). The first round of PCR was performed using *LA Taq* polymerase (TaKaRa) with the following protocol: 95°C for 3 min; 94°C for 30 s, after which the annealing temperature of the reaction was decreased by 2°C for 30 s every cycle, from 65°C to 55°C, followed by 72°C for 1 min; 25 cycles of 94°C for 30 s, 55°C for 30 s, and 72°C for 1 min; and final elongation at 72°C for 10 min. The primers used were UPM and 5RACE01. The final PCR volume was 25 μl containing approximately 50 ng cDNA, 0.2 mM of each dNTP, 1.5 mM of Mg^2+^, 0.2 μM of each primer, 2.5 μl of 10 × LA PCR buffer (Mg^2+^ free) and 0.25 μl (5 U/μl) of *LA Taq* polymerase (TaKaRa). The second round of PCR, also *LA Taq* polymerase (TaKaRa), was performed on 1 μl of the first round PCR product. The PCR conditions were: 95°C for 3 min; 30 cycles of 94°C for 30 s, 66°C for 30 s, and 72°C for 1 min; and final elongation at 72°C for 10 min. The primers used were NUP and 5RACE02. The final PCR volume was 25 μl and concentrations of all other reagents within the reaction were the same as the first round of PCR. Likewise, the synthesized first-stand 3′-RACE-ready cDNA was employed as a template to amplify the 3′ end of *CsuPLE1* cDNA using UPM and NUP and the two *CsuPLE1*-specific forward primers (3RACE01: 5′-GAAATGGTACATGCCGTTGCTCACAC-3′ and 3RACE02: 5′-TCATCTTGGCTTTCGCAGAAGGGTAG-3′). The conditions of these two rounds of PCR were the same as those for the amplication of the 5′ end of *CsuPLE1* cDNA, respectively. All the PCR products were subcloned into the pGEM-T Easy vector (Promega) and three clones were sequenced as described above.

### Vectorette PCR for TE-display and UPGMA tree construction

Vectorette PCR was used to isolate the *CsuPLE1*’s flanking sequences and to examine insertion site diversity. Vectorette PCR was performed as previously described [40]. Two anchoring bubble linker oligonucleotides were designed to make the vectorette unit for ligation to the *Hind* III digested gDNA.

The vectorette unit was prepared as described in Ko et al. [40]. For this analysis, eight individuals were randomly selected from each of 12 field populations. Approximately 1 μg of gDNA was digested at 37°C for 6 h using *Hind* III (NEB, Ipswich, MA, USA) in a 20 μl reaction. The digested gDNA was ligated with the vectorette unit using T4 DNA ligase (NEB). Two rounds of nested PCR with two pairs of primers were then carried out using the following primers: VPCR1: 5′-CCCTTCTCGAATCGTAACCG-3′ (vectorette external primer), VPCR2: 5′-CGTAACCGTTCGGTCCTCTG-3′ (vectorette internal primer), VP5R1: 5′-TGCCATGTCTGCAACGCACT-3′ (5′ specific external primer), VP5R2: 5′-AGCTGACACGTTTCTTACTGC-3′ (5′ specific internal primer). The Vectorette PCR was performed in a 10 μl reaction volume containing approximately 25 ng gDNA, 0.2 mM of each dNTP, 1.5 mM of Mg^2+^, 0.2 μM of each primer, 2.5 μl of 10 × LA PCR buffer (Mg^2+^ free) and 0.1 μl (5 U/μl) of *LA Taq* polymerase (TaKaRa). These two rounds of Vectorette PCR amplification conditions were 3 min at 95°C for initial denaturation, then 94°C for 30 s, after which the annealing temperature of the reaction was decreased by 1°C for 1 min every cycle, from 65°C to 51°C, followed by 72°C for 2 min 30 s; then 23 cycles of 94°C for 30 s, 52°C for 1 min, and 72°C for 2 min 30 s; and final elongation at 72°C for 10 min. For the second PCR, 1 μl of 100-fold diluted first round PCR product was used as the template. All the PCR products were visualised on a 2% agarose gel with ethidium bromide (EB) staining. To obtain the flanking sequences of *CsuPLE1* in *C. suppressalis* genome, the vectorette PCR products were cloned and sequenced as described above.

Based on the 5′ insertion sites of *CsuPLE1*, a 0 (no insertion) / 1 (with insertion) binary matrix was constructed. Genetic similarities (GS) (Additional file 8: Table S4) between pairs of field populations were measured as GS(*ij*) = 2*a*/(2*a* + *b* + *c*) where *a* is the number of co-existed insertion sites in both samples, *b* is the number of presence insertion sites in *i* but absent in *j*, and *c* is the number of presence insertion sites in *j* but absent in *i* [41,42]. Genetic similarities were used to construct a UPGMA phylogenetic tree in Phylip version 3.695 [43].

### The *CsuPLE1.1* insertion frequency, sequence variations and MP tree construction

Inverse PCR identified approximately 1480 bp of flanking sequence from the putative intact copy of *CsuPLE1.1*. The absence or presence of *CsuPLE1.1* in the insertion site (corresponding to the 5′ insertion site 2 in Table 1) in individual *C. suppressalis* was examined by flanking PCR with the primer pairs Flk-F (5′-TAACTAAGGTTCGCTGATGAC-3′) and Flk-R (5′-GATGCGCCTATCTATTTCG-3′). These primers flank the insertion site. For this analysis, a total of 945 individuals were randomly selected from 21 field populations. For each field populations, 45 individuals were selected (three groups, 15 individuals in each group). The absence of *CsuPLE1.1* at the insertion site was indicated by a 264 bp amplicon, and its presence was indicated by an approximately 2670 bp amplicon. The flanking PCR amplification conditions were as follows: initial denaturing at 94°C for 3 min, followed by 30 cycles of 94°C for 30 s, 55°C for 30 s, 72°C for 3 min, and a final extension at 72°C for 10 min. The PCR products were run on a 1.5% agarose gel with EB staining to detect the presence or absence of the *CsuPLE1.1* insertion in each individual. The resulting PCR products were purified, cloned and sequenced as described above.

To compare nucleotide variation inside and outside ORF regions, the *CsuPLE1.1* transposon sequence was divided into three parts: (i) the PLE 5′ outside the ORF region, (ii) inside the ORF region (from the initiator codon to the termination codon) and (iii) the PLE 3′ outside the ORF region. Four individuals containing the *CsuPLE1.1* copy in each of the 21 field populations were randomly selected from those samples mentioned in this section above and the *CsuPLE1.1* copy was purified, cloned and sequenced as described above. The four *CsuPLE1.1* copies were aligned independently for each of the 21 field populations using CLUSTAL X1.8. All nucleotide variation, including indels and single nucleotide polymorphisms, were scored as a single variant. The number of variants (*Nv*) and the length of each part (*L*) were used to calculate the variation rate (*Rv*) where *Rv* = *Nv*/*L*.

To examine variation in *CsuPLE1.1* sequences between field populations, all 84 *CsuPLE1.1* copies (4 *CsuPLE1.1* copies from 21 populations) were aligned using CLUSTAL X1.8, A phylogenetic tree was generated using Maximum Parsimony in MEGA 4.

## Additional files

Additional file 1: Figure S1. Gel analysis of the 5′ Vectorette PCR of CsuPLE1s in 21 populations. Additional file 2: Table S1. 5′ insertion sites and flanking sequences of CsuPLE1 in C. suppressalis. Additional file 3: Table S2. The viorations in CsuPLE1.1 copies. Additional file 4: Figure S2. The Variation hotspot in CsuPLE1.1 copies. Additional file 5: Figure S3. Phylogenetic tree constructed based on multiple sequence alignments of CsuPLE1.1 copies. Additional file 6: Figure S4. Phylogenetic tree constructed based on the 5′ insertion sites of CsuPLE1s. Additional file 7: Table S3. Longitude and latitude of sampling locations. Additional file 8: Table S4. The genetic similarities between pairs of field populations.

## Abbreviations

TE: Transposable element
PLE: *piggyBac*-like element
ITRs: Inverted terminal repeats
ORF: Open reading frame
TSDs: Target site duplications
NLS: Nuclear localization signal
UPGMA: Unweighted pair group method analysis
