# Supplementary material for: Molecular characterization of the piggyBac-like element, a candidate marker for phylogenetic research of Chilo suppressalis (Walker) in China
Source: BMC Mol Biol. 2014 Dec 17;15:28. doi: 10.1186/s12867-014-0028-y (PMC4273485; doi:10.1186/s12867-014-0028-y)
Supplement: Additional file 1: Figure S1. — Gel analysis of the 5′ Vectorette PCR of CsuPLE1s in 21 populations. [file 12867_2014_28_MOESM1_ESM.doc]

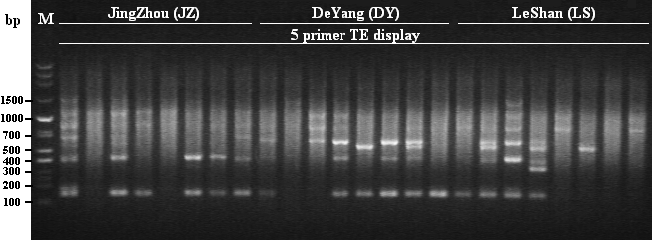

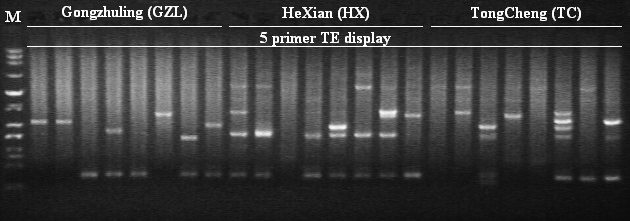


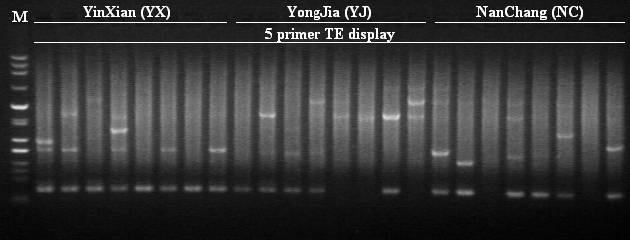

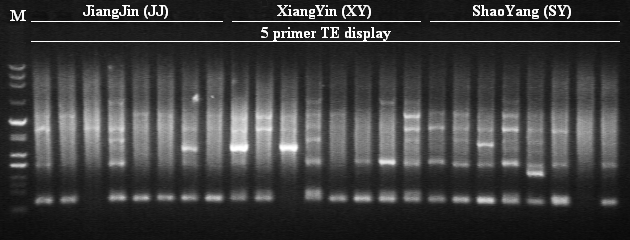


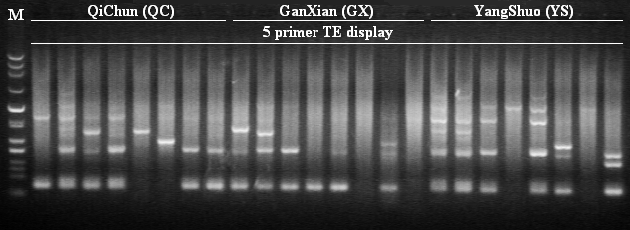

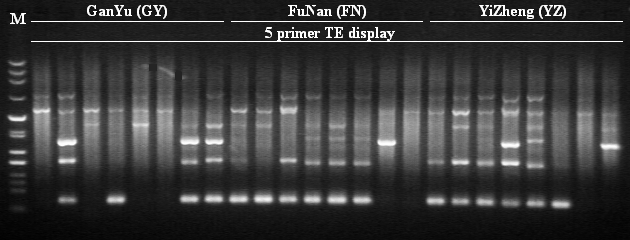


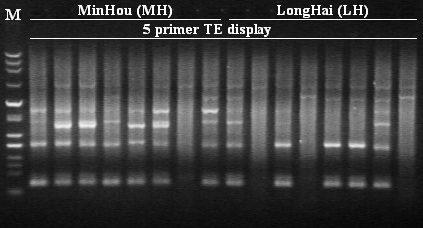

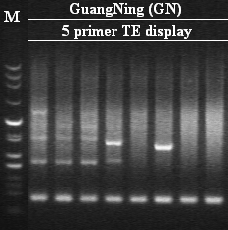


Figure S1. Gel analysis of the 5’ Vectorette PCR of *CsuPLE1*s in 21 populations. Eight individuals were randomly selected from each field population. The PCR products were displayed on 2% agarose gel. M indicates the DNA marker.
