# Supplementary material for: Molecular characterization of the piggyBac-like element, a candidate marker for phylogenetic research of Chilo suppressalis (Walker) in China
Source: BMC Mol Biol. 2014 Dec 17;15:28. doi: 10.1186/s12867-014-0028-y (PMC4273485; doi:10.1186/s12867-014-0028-y)
Supplement: Additional file 2: Table S1. — 5′ insertion sites and flanking sequences of CsuPLE1 in C. suppressalis. [file 12867_2014_28_MOESM2_ESM.doc]

Table S1 5’ insertion sites and flanking sequences of *CsuPLE1* in *C. suppressalis* genome

| Insertion site | Flanking sequence length (bp) | Insertion site sequence |
| --- | --- | --- |
| 1 | 11 | 5’-TCTGAGCTTCC |
| 2 | 228 | 5’-……CCACGAGTTGAATGAAGCTTCC |
| 3 | 777 | 5’-……CGTAACCTAACCTAACTTAATT |
| 4 | 1043 | 5’-……AAACATCAAGCAGTTACATGCT |
| 5 | 159 | 5’-……CACTTTTGTTTCCGGCCATCAGC |
| 6 | 1501 | 5’-……CACGAGGGTCTGATGTTCAAAC |
| 7 | 493 | 5’-……CAATCGGTTGAGGGCAAGTGCC |
| 8 | 43 | 5’-……CCGTTCGGTCCTCTGAGCTTCC |
| 9 | 516 | 5’-……CTTATTATATACCGGTGATCAT |
| 10 | 445 | 5’-……CTTTTATGTCACACACTTGAGA |
| 11 | 202 | 5’-……CAGCATTCTTCGGTACACGCAA |
| 12 | 26 | 5’-……AGCTTCCGTCGCGTGTGCAGCC |
| 13 | 124 | 5’-……CTTTACCGTCCTCTGAGCTTCC |
| 14 | 455 | 5’-……GCAATGTCAACAGATGAAAGTT |
| 15 | 175 | 5’-……TCGTGTTGTTGTATCTAGGGTA |
| 16 | 424 | 5’-……CTTATTATATA CCGGCGATCAT |
| 17 | 98 | 5’-……ATTAATGTTTTTTCTCACAGCC |
| 18 | 366 | 5’-……TATGTATTGAAGTAAACAAATA |
| 19 | 547 | 5’-……AGATAGTGTATGGCAGCTTATA |
| 20 | 530 | 5’-……TTCCAGTCAAAGTGACATTACA |
| 21 | 804 | 5’-……TCAATATTATACTGAAACCATA |

The omitted sequences are indicated by ellipses.
