# Supplementary figures and images for: Molecular characterization of the piggyBac-like element, a candidate marker for phylogenetic research of Chilo suppressalis (Walker) in China
Source: BMC Mol Biol. 2014 Dec 17;15:28. doi: 10.1186/s12867-014-0028-y (PMC4273485; doi:10.1186/s12867-014-0028-y)

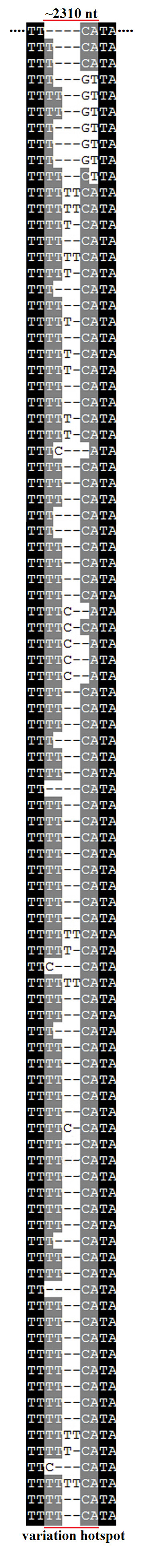

Supplement: Additional file 4: Figure S2. — The Variation hotspot in CsuPLE1.1 copies. [file 12867_2014_28_MOESM4_ESM.jpeg]
