# Supplementary material for: Molecular characterization of the piggyBac-like element, a candidate marker for phylogenetic research of Chilo suppressalis (Walker) in China
Source: BMC Mol Biol. 2014 Dec 17;15:28. doi: 10.1186/s12867-014-0028-y (PMC4273485; doi:10.1186/s12867-014-0028-y)
Supplement: Additional file 5: Figure S3. — Phylogenetic tree constructed based on multiple sequence alignments of CsuPLE1.1 copies. [file 12867_2014_28_MOESM5_ESM.doc]

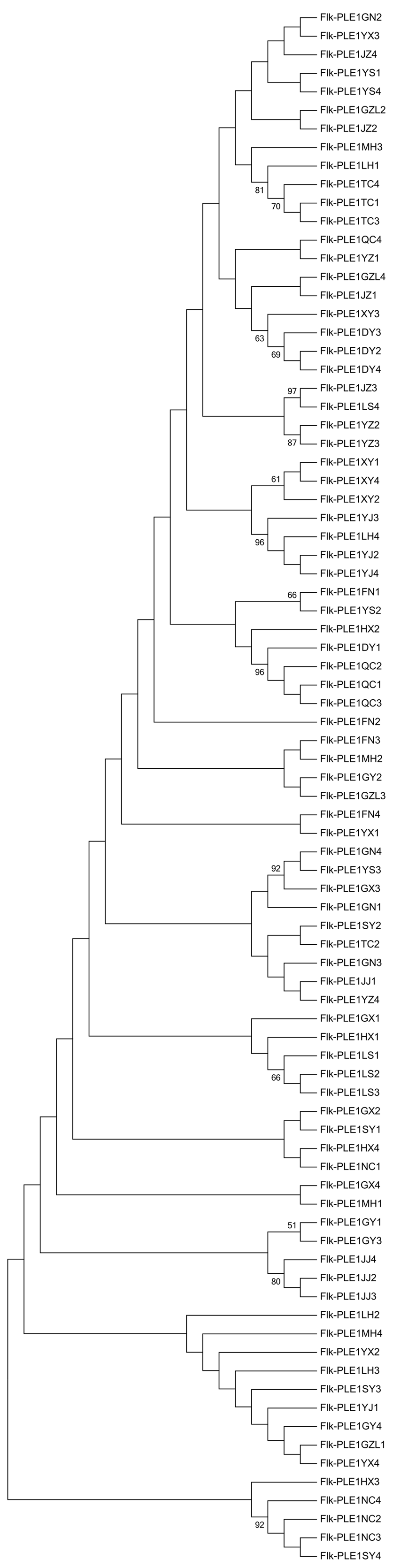


Figure S3. Phylogenetic tree constructed based on multiple sequence alignments of *CsuPLE1.1* copies. The tree was generated in MEGA4 using the Maximum Parsimony method with 1000 bootstrapping. Bootstrap values (> 50%) are shown at the nodes based on 1000 replications of the data set. Flk-PLE1YS1, 2, 3 and 4 are from the Yangshuo (YS) population. Flk-PLE1GX1, 2, 3 and 4 are from the Ganxian (GX) population, and so on.
