# Supplementary material for: Molecular characterization of the piggyBac-like element, a candidate marker for phylogenetic research of Chilo suppressalis (Walker) in China
Source: BMC Mol Biol. 2014 Dec 17;15:28. doi: 10.1186/s12867-014-0028-y (PMC4273485; doi:10.1186/s12867-014-0028-y)
Supplement: Additional file 6: Figure S4. — Phylogenetic tree constructed based on the 5′ insertion sites of CsuPLE1s. [file 12867_2014_28_MOESM6_ESM.doc]

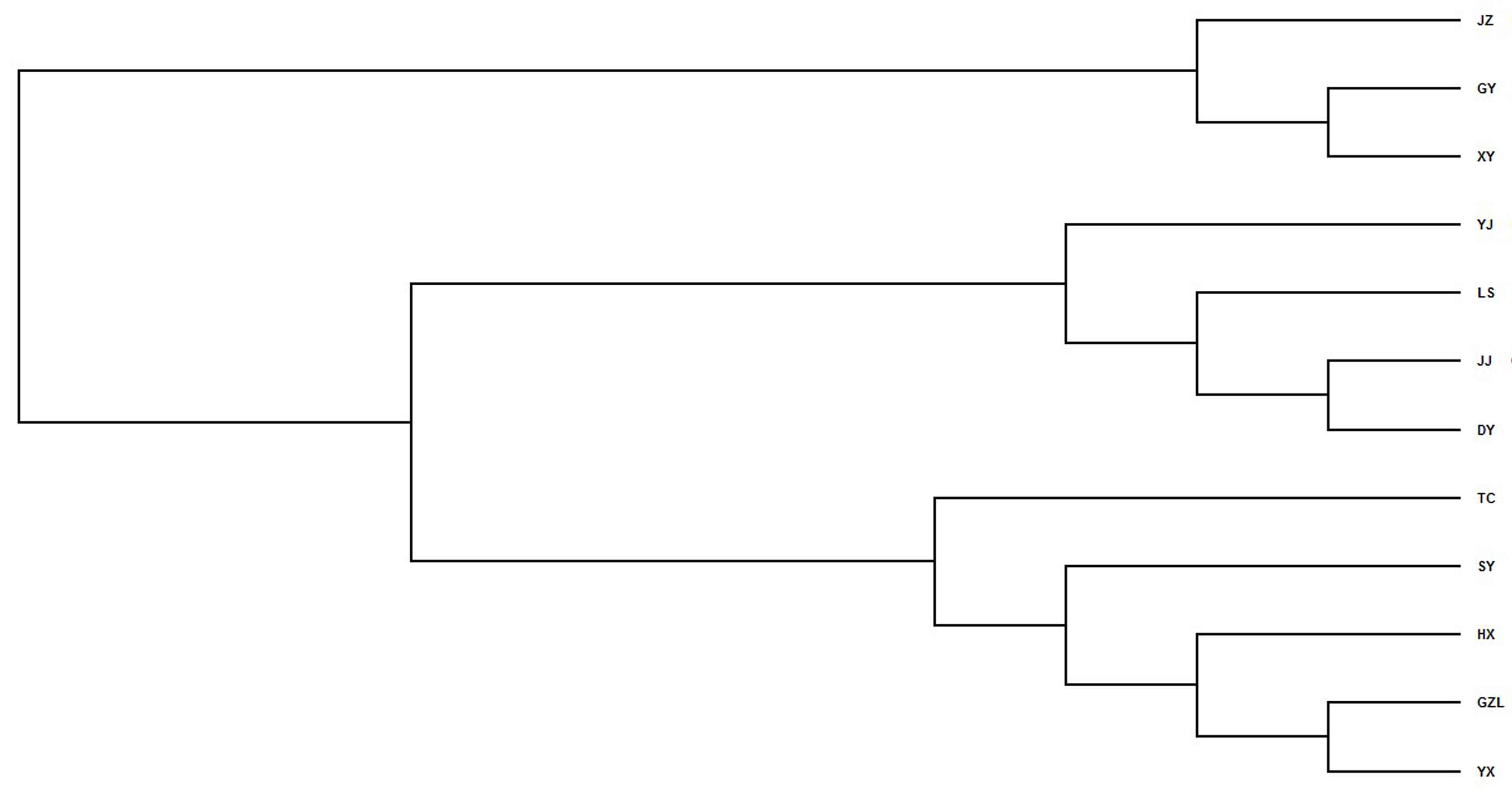


Figure S4. Phylogenetic tree constructed based on the 5’ insertion sites of *CsuPLE1s* (Table 1). The tree was generated in Phylip 3.695 using the UPGMA method. The letters are the location abbreviations.
