# Supplementary material for: Molecular characterization of the piggyBac-like element, a candidate marker for phylogenetic research of Chilo suppressalis (Walker) in China
Source: BMC Mol Biol. 2014 Dec 17;15:28. doi: 10.1186/s12867-014-0028-y (PMC4273485; doi:10.1186/s12867-014-0028-y)
Supplement: Additional file 7: Table S3. — Longitude and latitude of sampling locations. [file 12867_2014_28_MOESM7_ESM.doc]

Table S3 Longitude and latitude of sampling locations

| **Numbers** | **Sampling location** | **Longitude (E)** | **Latitude (N)** |
| --- | --- | --- | --- |
| 1 | GZL | 124:42:37 | 43:36:58 |
| 2 | GY | 119:08:53 | 34:47:32 |
| 3 | FN | 115:34:34 | 32:36:50 |
| 4 | YZ | 119:14:09 | 32:31:50 |
| 5 | HX | 118:21:25 | 31:43:03 |
| 6 | DY | 104:20:36 | 31:07:56 |
| 7 | TC | 116:53:25 | 30:57:23 |
| 8 | JZ | 112:20:29 | 30:16:48 |
| 9 | QC | 115:27:09 | 30:13:24 |
| 10 | YX | 121:31:43 | 29:48:26 |
| 11 | LS | 103:41:51 | 29:31:18 |
| 12 | JJ | 106:15:54 | 29:08:28 |
| 13 | XY | 112:54:28 | 28:38:45 |
| 14 | NC | 115:56:42 | 28:33:28 |
| 15 | YJ | 120:48:00 | 28:02:30 |
| 16 | SY | 111:28:10 | 27:17:36 |
| 17 | MH | 119:03:54 | 26:13:58 |
| 18 | GX | 115:02:03 | 25:56:10 |
| 19 | YS | 110:23:28 | 24:51:52 |
| 20 | LH | 117:50:06 | 24:27:15 |
| 21 | GN | 112:25:46 | 23:35:52 |
