# Supplementary material for: Molecular characterization of the piggyBac-like element, a candidate marker for phylogenetic research of Chilo suppressalis (Walker) in China
Source: BMC Mol Biol. 2014 Dec 17;15:28. doi: 10.1186/s12867-014-0028-y (PMC4273485; doi:10.1186/s12867-014-0028-y)
Supplement: Additional file 8: Table S4. — The genetic similarities between pairs of field populations. [file 12867_2014_28_MOESM8_ESM.doc]

Table S4 The genetic similarities between pairs of field populations

| Sampling  locations | Genetic similarities (GS) | | | | | | | | | | | |
| --- | --- | --- | --- | --- | --- | --- | --- | --- | --- | --- | --- | --- |
|  | **1** | **2** | **3** | **4** | **5** | **6** | **7** | **8** | **9** | **10** | **11** | **12** |
| **1.GY** | 0 |  |  |  |  |  |  |  |  |  |  |  |
| **2.JJ** | 0.7143 | 0 |  |  |  |  |  |  |  |  |  |  |
| **3.XY** | 0.5000 | 0.6000 | 0 |  |  |  |  |  |  |  |  |  |
| **4.SY** | 0.4286 | 0.5000 | 0.6000 | 0 |  |  |  |  |  |  |  |  |
| **5.JZ** | 0.5000 | 0.6000 | 0.7500 | 0.6000 | 0 |  |  |  |  |  |  |  |
| **6.DY** | 0.6667 | 0.7692 | 0.5455 | 0.4615 | 0.5455 | 0 |  |  |  |  |  |  |
| **7.LS** | 0.5000 | 0.5714 | 0.3333 | 0.2857 | 0.5000 | 0.6667 | 0 |  |  |  |  |  |
| **8.GZL** | 0.5333 | 0.4615 | 0.5455 | 0.6154 | 0.5455 | 0.5714 | 0.5333 | 0 |  |  |  |  |
| **9.HX** | 0.6667 | 0.6154 | 0.5455 | 0.4615 | 0.5455 | 0.5714 | 0.4000 | 0.7143 | 0 |  |  |  |
| **10.TC** | 0.4615 | 0.3636 | 0.4444 | 0.3636 | 0.6667 | 0.5000 | 0.6154 | 0.5000 | 0.5000 | 0 |  |  |
| **11.YJ** | 0.5714 | 0.6667 | 0.4000 | 0.3333 | 0.6000 | 0.4615 | 0.5714 | 0.3077 | 0.4615 | 0.5455 | 0 |  |
| **12.YX** | 0.6667 | 0.6000 | 0.7500 | 0.6000 | 0.7500 | 0.7273 | 0.5000 | 0.7273 | 0.7273 | 0.6667 | 0.4000 | 0 |
